# Prognostic factors for metastatic cutaneous squamous cell carcinoma of the parotid

**DOI:** 10.1186/1916-0216-42-14

**Published:** 2013-02-05

**Authors:** Fawaz M Makki, Adrian I Mendez, S Mark Taylor, Jonathan Trites, Martin Bullock, Gordon Flowerdew, Robert D Hart

**Affiliations:** 1 Department of Surgery, Division of Otolaryngology, Queen Elizabeth II Health Sciences Centre and Dalhousie University, 1278 Tower Rd, Halifax, NS, B3H 2Y9, Canada; 2 Dalhousie University, Anatomical Pathology, Halifax, Nova Scotia, Canada; 3 Department of Community Health & Epidemiology, Dalhousie University, Halifax, Nova Scotia, Canada

**Keywords:** Parotid gland, Squamous cell carcinoma, Metastasis

## Abstract

**Objective:**

To explore the prognostic significance of patient and disease characteristics on the survival of patients with metastatic cutaneous squamous cell carcinoma of the parotid gland at a tertiary care center in Halifax, Nova Scotia, Canada.

**Methods:**

A retrospective chart review for all patients diagnosed with metastatic cutaneous squamous cell carcinoma to the parotid gland from January 2000 to December 2010. Multiple variables were examined related to: patient demographics, surgical details, non-surgical procedure details, and tumor pathologic description.

**Results:**

A total of 54 patients [48 men (88%) and 6 women (12%)], with a median age at surgery of 78 years (range 47–93 years) were included in the study. All patients had a minimum follow up of 12 months or until deceased, with a median duration of follow up of 24 months. Predictors that were significant for cancer recurrence were pretreatment N-stage, pathologic neck node status, total number of positive neck nodes, and perineural invasion. Predictors that were significant for cancer death were the total number of positive neck nodes and perineural invasion. The remainder of the predictors including margin status were non-significant. Only age and nodal status were significant for both cancer death and recurrence on multivariate analysis.

**Conclusion:**

Our results showed only two variables that remained significant on multivariate analysis were age and number of involved neck nodes, this finding suggests that re-resection of positive margins may not be necessary and that radiation therapy is the mainstay of treatment for positive margins.

## Introduction

The parotid gland is anectodermal derivative. During the development of the ectoderm, the parotid gland harbors three groups of lymph nodes. The first group is embedded within the parotid fascia, the second is in the parotid parenchyma, and the third remains extrafascial and extraglandular (ex: periauricular). Parotid cancers are relatively uncommon, representing fewer than 5% of all head and neck (H&N) cancers [1,2]. In the majority of cases, the pathological origin is either primary parotid malignancy or parotid metastasis arising from a cutaneous malignancy of the H&N. Squamous cell carcinoma (SCC) is the second most common subtype of skin cancer after basal cell carcinoma and accounts for 20% of all nonmelanotic skin cancers in the head and neck area [3]. The incidence of metastatic cutaneous SCC of the H&N is low (≈ 5%) [4,5]. Lymphatic spread of H&N cutaneous SCC often first involves parotid and subsequently cervical area lymph nodes [6]. The presence of nodal metastasis, especially to the parotid lymph nodes, is an indicator of poor prognosis [7].

There are two different staging systems for metastatic cutaneous SCC of the parotid: 1) the American Joint Committee on Cancer (AJCC) and 2) the O’Brien staging system. The AJCC has been criticized as an inadequate staging system for cutaneous SCC. In this system all patients with regional metastasis are designated N1, thereby failing to reflect the extent of parotid gland and cervical lymph node involvement. In 2002, O’Brien et al. [8] developed a staging system that separates parotid and neck node involvement into (P) and (N) stages (Table 1). A multicenter study has confirmed the superiority of the O’Brien staging system to the AJCC as a prognostic indicator of patients with metastatic cutaneous SCC of the parotid gland [9].

**Table 1:** O’Brien et al. system for clinical staging of metastatic cutaneous SCC involving the parotid gland

**O’Brien et al. system for clinical staging of metastatic cutaneous SCC involving the parotid gland **[8]	
**Parotid**	
P1	Metastatic node ≤ 3 cm
P2	Metastatic node > 3 cm but ≤ 6 cm or
	Multiple parotid nodes
P3	Metastatic node > 6 cm or
	Disease involving facial nerve or skull base
**Neck**	
N0	No clinical neck disease
N1	Single ipsilateral neck node ≤ 3 cm
N2	Single node > 3 cm or
	Multiple nodes or Contralateral neck nodes

There is considerable interest in determining predictors of patient outcome in metastatic cutaneous SCC of the parotid. Variables include patient demographics (ex: age, sex), pathology (ex: degree of differentiation, cervical node involvement, intraparotid node involvement, perineural invasion), and treatment types (ex: surgery alone, radiation therapy alone, surgery and radiation therapy, type of parotidectomy, facial nerve preservation). Combined treatment with surgery and radiation therapy is the optimum treatment modality and standard of care for metastatic cutaneous SCC of the H&N [10,11]. Conversely, evidence of facial nerve involvement, positive margins, and surgery alone without postoperative radiotherapy have been previously identified as indicators of poor prognosis [6,12,13].

In this study, our primary aim was to evaluate predictors of treatment outcome (local recurrence and disease-specific survival), including effect of margin status (positive, narrow (≤ 5 mm), negative). Our second aim was to review our experience with metastatic cutaneous SCC to the parotid at the Queen Elizabeth II hospital in Halifax, Nova Scotia, Canada.

## Methods

A retrospective chart review was performed to identify all patients diagnosed with metastatic cutaneous SCC to the parotid gland from January 2000 to December 2010. Hospital research ethics board committee approval was obtained. Patients were identified by a medical record search of the Capital district health authority and of Cancer Care Nova Scotia. We included all patients aged 18 years and above with pathology proven diagnosis of metastatic cutaneous SCC to the parotid, having a minimum follow-up of one year, a complete pathology report including the status of both deep and superficial margins, and that were treated with curative intent.

All patients were discussed and assessed in multidisciplinary head and neck tumor rounds. Treatment options included either surgery alone or surgery and radiation therapy (XRT) depending on multiple factors including tumor margin status, neck node status, and patients’ general medical health. Surgical intervention ranged from a simple superficial parotidectomy with facial nerve sparing without neck dissection to a total parotidectomy with facial nerve resection and neck dissection. Parotidectomy with facial nerve sparing was defined as complete or partial preservation, allowing for a few branches to be sacrificed as they were either encased by tumor or demonstrated paralysis preoperatively.

**Figure 1:**
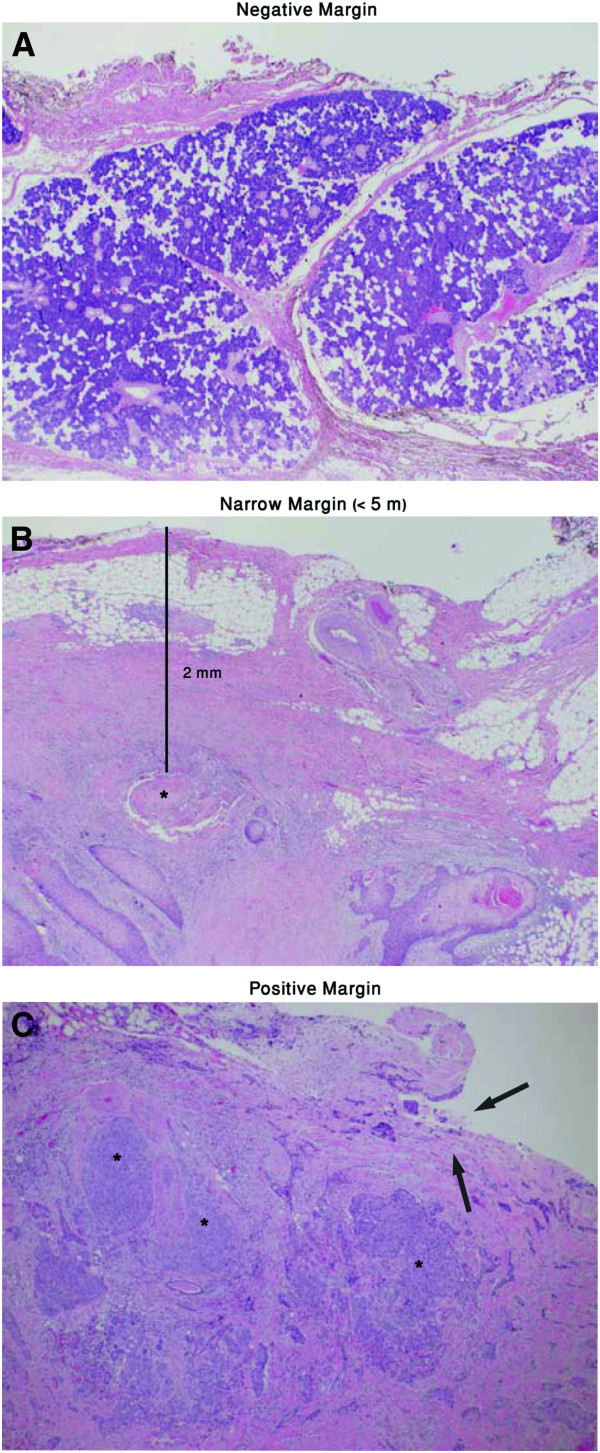
**A: Cauterized parotid tissue with inked surgical margin in upper part of field.** No tumor is present in this image. **B:** Fibrotic parotid tissue containing nests of invasive keratinizing squamous cell carcinoma as close as 2 mm from the surgical margin. **C:** Poorly differentiated squamous cell carcinoma replacing most of the tissue, with involvement of the deep tissue margin (arrows).

Data collected included patients demographics (ex: age, sex), treatment details (ex: surgery only, surgery and XRT or XRT only), procedure details (type of parotidectomy, facial nerve preservation, neck dissection), tumor characteristics (ex: side, size), pathological description (ex: degree of differentiation, extra-parotid extension, perineural invasion, lymphovascular invasion, margin status both superficial and deep, and neck node status, number of positive neck nodes, intraparotid lymph node status ,and number of positive intraparotid lymph nodes), and outcome data (recurrence, time to recurrence, death, time to death, and cause of death). For each patient two surgical margins were analyzed, superficial and deep. Each margin was reported as negative, narrow (≤ 5 mm) or positive (Figure 1). In addition to the superficial and deep margin status, we defined the combined margin status to be whichever was worse. For example, if a patient had a positive deep margin and a negative superficial margin then the combined margin status was positive. For staging purposes, the O’Brien staging system was used in this series.

The data were analyzed using the Cox proportional hazards program (PHREG) in SAS version 9.2 (SAS Institute, Cary, NC). The following variables were investigated for their association with time to cancer recurrence and time to cancer death: age, sex, diagnosis, parotidectomy, facial nerve preservation, neck dissection, P-stage, N-stage, tumor dimension, neck node status (binary and 5-point scale [Table 2]), intraparotid lymph node status (binary and 5-point scale [Table 2]), extraparotid extension, perineural invasion, lymphovascular invasion, superficial margin status, deep margin status, combined margin status and radiation therapy. Univariate and stepwise multivariate approaches were used to determine the prognostic variables. Age and the two 5-point scales were treated as continuous variables in these analyses. Hazard ratios with 95% confidence intervals and associated p-values were calculated. We report the results only for variables with *p* ≤ 0.10. Kaplan-Meier survival curves were constructed showing the association between key variables and time to cancer recurrence and cancer death. Maximum likelihood estimates of the average times to recurrence and disease specific death (M) were obtained under the constant hazard assumption by dividing the total person-time of follow-up by the number of outcome events.

**Table 2:** Five-point scale for positive neck nodes and intraparotid lymph nodes

**Number of positive nodes**	**5 point scale**
0	0
1	1
2	2
3	3
4+	4

## Results

All results are summarized in Tables 3 and 4.

**Table 3:** Univariate analysis for cancer death and recurrence

**Variables**	**Cancer death**			**Recurrence**		
	**Hazard ratio**	**95% CI**	***p***	**Hazard ratio**	**95% CI**	***p***
**Sex**	- *	-	-	-	-	-
**Age**	.94 per year of age	.89-.99	.*009*	.96 per year of age	.93-1.01	.064
**Degree of differentiation (well, moderate, poor)**	-	-	-	-	-	-
**Type of parotidectomy** **(superficial v. total)**	-	-	-	-	-	-
**Facial nerve preservation (yes *v. *no)**	-	-	-	-	-	-
**Neck dissection (yes *v. *no)**	-	-	-	-	-	-
**P - Stage (1, 2, 3)**	2.59 per unit increase	.93-8.33	.067	1.97	.93-4.38	.075
**N - Stage (0, 1, 2)**	1.85 per unit increase	.95-3.58	.069	1.92	1.17-3.11	*.011*
**Tumor dimension (cm)**	1.37	.99-1.91	.055	1.24	.98-1.58	.072
**Neck node status (yes *v. *no)**	2.70	.75-9.73	.124	3.20	1.24-8.24	.*017*
**Neck node status (5 point scale)**	1.46 per unit increase	1.02-2.05	.*039*	1.49 per unit increase	1.13-1.92	.*006*
**Intraparotid LN status (yes *v. *no)**	-	-	-	-	-	-
**Intraparotid LN status (5 point scale)**	-	-	-	-	-	-
**Extraparotid extension (yes *v. *no)**	-	-	-	-	-	-
**Perineural invasion (yes *v. *no)**	5.76	1.08-106.3	.*039*	3.26	1.07-14.1	.*036*
**Lymphovascular invasion (yes *v. *no)**	-	-	-	-	-	-
**Superficial margin status (negative, narrow, positive)**	-	-	-	-	-	-
**Deep margin status (negative, narrow, positive)**	-	-	-	-	-	-
**Combined margin status (negative, narrow, positive)**	-	-	-	1.82	.95-4.07	.073
**Radiation therapy (yes *v. *no)**	-	-	-	-	-	-

* Hyphens (−) were inserted in the table when p>.1.

**Table 4:** Multivariate analysis for cancer death and recurrence

**Predictor**	**Death from cancer**			**Recurrence**		
	**Hazard ratio**	**95% CI**	***p***	**Hazard ratio**	**95% CI**	***p***
**Age**	.92 per year of age	.86-.97	.002	.95 per year of age	.91-.99	.014
**Nodal status (5 point scale)**	1.72 Per unit increase	1.19-2.44	.003	1.58 Per unit increase	1.20-2.05	.001

### Study population

The study population included a total of 54 patients [48 men (88%) and 6 women (12%)], with a median age at surgery of 78 years (range 47–93 years) and a mean age of 75 years. All patients had a minimum follow up of 12 month or until deceased, with a median duration of follow up of 24 months. All patients had a prior history of cutaneous SCC of the head and neck. Both neck and parotid disease were staged using the O’Brien staging system (Table 1).

### Treatment

All patients had parotid surgery for their metastatic cutaneous SCC, 34 patients (63%) underwent superficial parotidectomy and 20 patients (37%) underwent total parotidectomy. The facial nerve was either fully or partially preserved in 41 patients (76%) and sacrificed in 13 patients (24%). Forty-nine patients underwent neck dissection (91%). Radiation therapy was provided to 47 patients (87%) In the remaining seven patients, five patients did not require radiation therapy and two patients refused it.

### Pathology of metastatic cutaneous SCC

SCC was well differentiated in 3 patients (5%), moderately differentiated in 28 patients (52%), and poorly differentiated in 23 patients (43%). The median size of the parotid metastasis was 4.0 cm (range: 1.0-8.1 cm). Sixteen patients (30%) had clinically positive neck nodes, while 38 patients (70%) had no evidence of neck disease. In terms of intraparotid lymph nodes, 30 patients (56%) had only a single node involved and 24 patients (44%) had multiple intraparotid nodes affected. Neck disease was staged as N0 in 38 patients (70%), N1 in 3 patients (6%), and N2 in 13 patients (24%). Parotid disease was staged as P1 in 9 patients (16%), P2 in 29 patients (54%), and P3 in 16 patients (30%). Extraparotid extension was positive in 45 patients (83%). Perineural invasion was positive in 35 patients (65%). Lymphovascular invasion was positive in 15 patients (28%). Margin status is summarized in Table 5.

**Table 5:** Margin status distribution summary

	**Negative**	**Narrow (< 5 mm)**	**Positive**	**Total**
**Superficial M.**	36	14	4	54
**Deep M.**	13	13	28	54
**Combined M.**	12	13	29	54

### Outcome data

**Figure 2:**
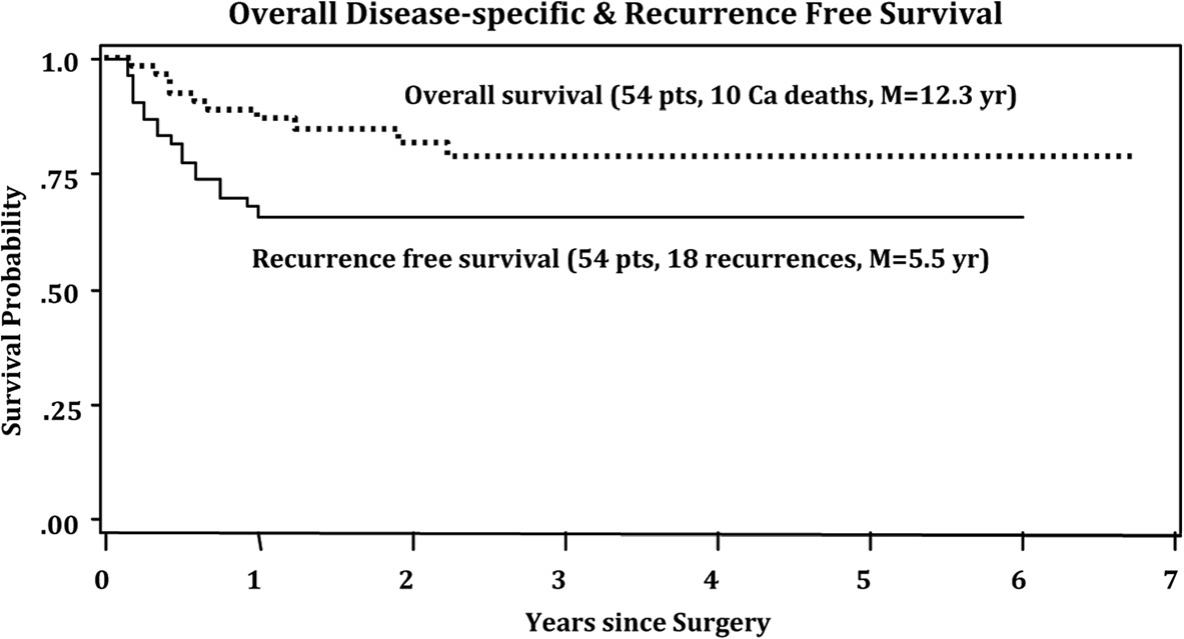
Overall disease-specific and recurrence free survival.

The overall recurrence rate was 33% (18 of 54). Median time to recurrence was 4.5 months (range 1.5-12 months) (Figure 2). All recurrences were in patients who had both surgery and radiation therapy, except 2 patients who refused radiation therapy after surgery. The 2-year recurrence free survival rate was 60% (32 of 54 patients). The two-year overall survival rate was 70% (38 of 54 patients) and the two-year disease specific survival rate was 81% (44 of 54 patients). The total number of deaths were 16 (30%), 10 deaths (19%) were cancer related and 6 deaths (11%) were non-cancer related (Figure 2).

**Figure 3:**
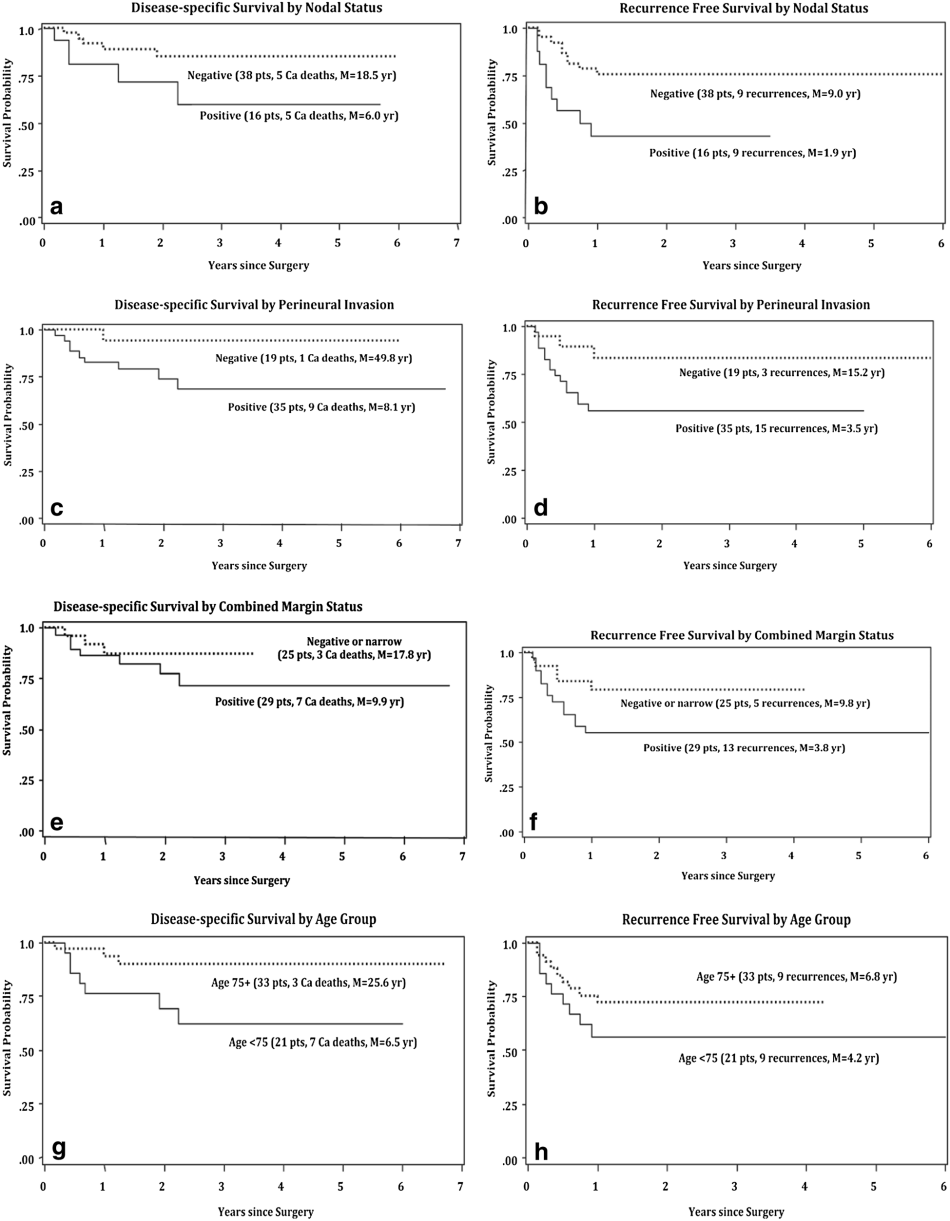
**a: Disease-specific survival by nodal status. b:** Recurrence free survival by nodal status. **c:** Disease-specific survival by perineural invasion. **d:** Recurrence free survival by perineural invasion. **e:** Disease-specific survival by combined margin status. **f:** Recurrence free survival by combined margin status. **g:** Disease-specific survival by age group. **h:** Recurrence free survival by age group.

On univariate analysis, variables that were significant for *recurrence* were the presence of neck disease (*p* = 0.017) (Figure 3b), higher N staging for neck disease (*p* = 0.011), and the presence of perineural invasion (*p* = 0.036) (Figure 3d). Subdivision of patients into 5 groups (Table 2) based on the number of positive nodes (0, 1, 2, 3, 4+) demonstrated higher recurrence with increased nodal involvement (*p* = 0.006). In patients with negative neck nodes the estimated average time to recurrence (M) was 9 years, whereas in patients with positive neck nodes was 1.9 years (Figure 3b). For perineural invasion the average time to recurrence (M) was 3.5 years in patients with positive perineural invasion and 15.2 years in patients were perineural invasion was absent (Figure 3d). All other variables including margin status (superficial, deep and combined) (Figure 3f) were not statistically significant for recurrence.

On the other hand, variables that were significant on univariate analysis for *cancer death* were the presence of perineural invasion (*p =* 0.039) (Figure 3c) and increased number of positive neck nodes (*p* = 0.039). The average time to disease-specific death (M) was 8.1 years with positive perineural invasion and 49.8 years in the absence of perineural invasion. Our data also showed a decrease in cancer death in the elderly (*p* = 0.009) (Figure 3g). The average time to disease-specific death (M) was 25.6 years in patients above 75 years of age and 6.5 years in patients below the age of 75 years. The other variables, including margin status (superficial, deep, and combined) (Figure 3e) and P stage were not statistically significant for recurrence or cancer death on univariate analysis.

Only age and neck nodes status were significant on multivariate analysis. Older patients and those with fewer positive neck nodes had significantly better prognosis than younger patients and those with a higher number of positive nodes. No other variable met the 0.1 (or even 0.2) significance level for entry into the model (Table 4).

## Discussion

Sun exposed areas of the head and neck are some of the most common involved sites with cutaneous malignancy (squamous cell carcinoma, basal cell carcinoma, and melanoma). Although metastases from cutaneous SCC are uncommon, their presence especially in neck and/or intraparotid lymph nodes is an indicator of aggressiveness and poor prognosis. The relatively low frequency of metastatic spread of cutaneous SCC to the parotid and or neck lymph nodes is the main obstacle in being able to perform a retrospective study investigating the effect of certain variables on patient outcome. The highest incidence of cutaneous squamous cell carcinoma (SCC) in the world is in Australia with an annual incidence of over 300 cases per 100 000 people [14]. In this retrospective study we analyzed data on all patients operated in our institution with metastatic cutaneous SCC to the parotid and treated with curative intent. We also analyzed the impact of several predictors on the rates of recurrence and cancer deaths. Several factors have been shown previously to predict local recurrence such as involved margins [15], poorly differentiated SCC, diameter > 2 cm, depth > 4 mm, perineural invasion, and absence of adjuvant radiotherapy [6].

In our series, most patients were treated with both surgery and radiation therapy. Only 7 out of the 54 patients did not get radiation therapy. In 5 patients radiation therapy was not indicated (negative margin, no neck node involvement, negative perineural invasion, negative lymphovascular invasion and metastatic parotid node is ≤ 3 cm) and none of these patients had recurrence. The other 2 patients refused radiation therapy despite having involved margins and positive neck nodes. Studies have proven that metastatic cutaneous SCC to the parotid is best treated with surgery and postoperative radiation therapy [10-12].

In this retrospective study we did investigate the effect of multiple predictors on local recurrence and cancer death. Intraoperative margins were sent to the pathologist labeled as either superficial or deep margins. Superficial margins can be include either skin margins if the tumor involves the skin or from the most superolateral margin of the tumor resection. Deep margin are principally from the tumor resection bed. Margin status was reported as negative, positive, or narrow if within 5 mm. There was no significant difference in local recurrence or cancer death rates when comparing patients with positive margins to others with either negative or narrow margins. The combined margin status category, which is the worst status of either the deep or superficial margin, was also not statistically significant. However looking at the rates of each margin status in Table 6 there is an increase in the rates of recurrence and cancer death when comparing negative, narrow, and positive margins. This is most likely due to the relatively small patient numbers in each group our data didn’t reach statistical significance. In 1995, Khurana et al. has noted that positive surgical margins were associated with poorer local disease control (P < 0.05). Some recent studies demonstrated that positive margins do increase in the rate of recurrence [12], while others refuted this [13]. However, all previously published studies are in agreement that postoperative radiation therapy decreases the rate of recurrence in patients with involved margins [12,13].

**Table 6:** The number and percentage of each margin status based on patient status

**Combined margin status**	**Disease status**		
	**Alive & No disease**	**Alive with recurrence**	**Cancer death**
**Negative (12) (22%)**	10 (84%)	1 (8%)	1 (8%)
**Narrow (13) (24%)**	7 (54%)	4 (30%)	2 (16%)
**Positive (29) (54%)**	9 (31%)	13 (45%)	7 (24%)
**Total (54) (100%)**	26	18	10

The O’Brien staging system was used in this study rather than the AJCC staging system as it has been proven to be superior in predicting patient prognosis (Table 1). The P- and N- stages did not show statistical significance in the rates of cancer death, p-value at 0.067 and 0.069 respectively. In terms of recurrence rates, N-stage was statistically significant, p-value 0.011, but not P-stage. In 2008, Hinerman et al. showed statistically significant difference in local and loco-regional control and disease free survival when separated by clinical P-stage but not N-stage [12]. We also investigated the effect of an increase in number of positive neck or intraparotid lymph nodes as a continuous variable, and that did show significant increase in the rate of both cancer death (*p* = 0.039) and recurrence (*p* = 0.006) with an increasing number of positive neck nodes, but not intraparotid nodes. Kelder et al. [16] did similar analysis investigating the effect of increase in the number of positive lymph nodes on overall survival and disease free survival, which was significant with a *p* value of < 0.001.

Perineural invasion on univariate analysis was associated with a significant increase in both the rate of cancer death and recurrence. However, the effect of the other pathological variables (ex: lymphovascular invasion and extraparotid extension) did not show any significance neither in cancer death or recurrence. In our analysis, elderly patients were associated with significant decrease in the rate of cancer death (p = 0.009) (Figure 3g). The average time to disease-specific death (M) was 25.6 years in patients above 75 years of age and 6.5 years in patients below the age of 75 years. This might be an incidental finding or might correlate with a less aggressive in elderly. The only two variables that remained significant on multivariate analysis were age and number of involved neck nodes, which showed that older patients and those with fewer neck nodes involvements had significantly better prognosis than younger patients and those with a higher number of positive nodes for both disease-specific survival and recurrence free survival.

## Conclusion

Metastatic cutaneous SCC to the parotid is an aggressive disease. Prognosis is poor and ranges between 30% and 75% [17,18]. Neck disease, higher N-stage, larger number of positive neck nodes, and perineural invasion were associated with higher risk of recurrence. The risk of cancer death was higher with perineral invasion, higher number of positive neck nodes and patients younger than 75 years of age.

Despite previous reports on margin status affecting outcomes in metastatic SCC of the parotid, this study found the only two variables that remained significant on multivariate analysis were age and number of involved neck nodes, This finding suggests that re-resection of positive margins may not be necessary and that radiation therapy is the mainstay of treatment for positive margins.

## Competing interests

The authors declare that they have no competing interests.

## Authors' contributions

FM: Data collection and manuscript writing. AM:Data collection. SMT: Staff head & neck surgeon. JT: Staff head & neck surgeon. MB: Pathologist. GF:Statistician. RH:Senior author and Staff head & neck surgeon. All authors read and approved the final manuscript.
